# Patient and social factors related to nebulizer use in COPD patients at the transition of care: a qualitative study

**DOI:** 10.1186/s12890-023-02651-w

**Published:** 2023-09-23

**Authors:** Amanda A. Foster, Jennifer Stoll, Christopher J. Daly, Collin M. Clark, Sanjay Sethi, David M. Jacobs

**Affiliations:** 1 Department of Pharmacy Practice, Wegmans School of Pharmacy, St. John Fisher University, Rochester, NY USA; 2 Department of Family Medicine, Jacobs School of Medicine and Biomedical Sciences, University at Buffalo, Buffalo, NY USA; 3 Department of Pharmacy Practice, School of Pharmacy and Pharmaceutical Sciences, University at Buffalo, 316 Pharmacy Building, Buffalo, NY 14214 USA; 4 Department of Pulmonary, Critical Care, and Sleep Medicine, Jacobs School of Medicine and Biomedical Sciences, University at Buffalo, Buffalo, NY USA

**Keywords:** COPD, Social factors, Patient factors, Nebulizer therapy, Transitions of care, Qualitative research

## Abstract

**Background:**

Transition from hospital to home is a vulnerable period for patients with COPD exacerbations, with a high risk for readmission and mortality. Twenty percent of patients with an initial hospitalization for a COPD exacerbation are readmitted to a hospital within 30 days, costing the health care system over $15 billion annually. While nebulizer therapy directed at some high-risk COPD patients may improve the transition from hospital to home, patient and social factors are likely to contribute to difficulties with their use. Current literature describing the COPD patient’s experience with utilizing nebulizer therapy, particularly during care transitions, is limited. Therefore, the objective of this study was to explore underlying COPD patient and social factors contributing to practical difficulties with nebulizer use at the care transition from hospital to home.

**Methods:**

This was a qualitative study conducted between September 2020 and June 2022. Patients were included if they were ≥ 40 years old, had a current diagnosis of COPD, had an inpatient admission at a hospital, and were discharged directly to home with nebulizer therapy. Semi-structured, one-on-one interviews with patients were conducted covering a broad range of patient and social factors and their relationships with nebulizer use and readmission. Interviews were recorded and transcribed verbatim. A thematic analysis was performed using a mixed inductive and deductive approach.

**Results:**

Twenty-one interviews were conducted, and subjects had a mean age of 64 ± 8.4 years, 62% were female, and 76% were White. The predominant interview themes were health care system interactions and medication management. The interviews highlighted that discharge counseling methods and depth of counseling from hospitals were inconsistent and were not always patient-friendly. They also suggested that patients could appropriately identify, set up, and utilize their nebulizer treatment without difficulties, but additional patient education is required for nebulizer clean up and maintenance.

**Conclusions:**

Our interviews suggest that there is room for improvement within the health care system for providing consistent, effective discharge counseling. Also, COPD patients discharged from a hospital on nebulizer therapy can access and understand their treatment but require additional education for nebulizer clean up and maintenance.

**Supplementary Information:**

The online version contains supplementary material available at 10.1186/s12890-023-02651-w.

## Introduction

Chronic obstructive pulmonary disease (COPD) affects one in twenty American adults and costs the health care system more than $50 billion annually [1, 2]. COPD exacerbations are responsible for over 873,000 emergency room visits and 700,000 hospital admissions each year [1]. Furthermore, one in every five patients with an initial hospitalization for a COPD exacerbation is readmitted to the hospital within 30 days, costing the health care system over $15 billion annually [1]. Thus, there is considerable interest in identifying factors related to readmission and improving a COPD patient’s transition from hospital to home.

Adherence to inhaled therapies is a significant risk factor for hospitalization or readmission in COPD patients [3, 4]. Inhaled COPD treatments are primarily delivered through pressurized metered-dose inhalers (pMDIs), dry powder inhalers (DPIs), or nebulizers [5]. Nebulizer-based therapy may be more valuable for older adult patients or those who find it difficult to use hand-held inhalers (e.g. – pMDIs, DPIs), require high treatment doses, or have multiple comorbidities [6, 7]. Nebulizers overcome some of the limitations of pMDIs and DPIs, such as user error, and better deliver the drug allowing a greater margin of user error to still receive a therapeutic dose [6, 8, 9]. However, up to 50% of patients on nebulizer therapy experience practical difficulties with their nebulizer or do not receive proper instruction on its use [10, 11]. Pertinent patient or social factors such as social support, health literacy, and access to health care may interact with optimal nebulizer therapy use and therefore impact readmission risk.

While many medical factors, including medication adherence rates, are associated with hospital readmissions for COPD patients, several demographic and socioeconomic factors have also been explored. For example, demographic factors such as age ≥ 45 years, being identified as male at birth, and being White increase the probability of being readmitted for a COPD exacerbation, while economic factors include being a Medicaid recipient and residing in the lowest income areas [12]. Social factors that increase risk of readmission include being single (versus having a supporting spouse), living in a large metropolitan area with at least one million residents, and exposure to passive smoking [12–14]. These sociodemographic characteristics are limited to discreet variables commonly referred to as Z-codes that may be found within electronic health records (EHRs) or administrative databases [15, 16]. To identify higher-level socioeconomic and social environmental factors related to readmission, additional data collection strategies are needed such as qualitative interviews or mixed method approaches. However, these approaches can be time and resource intensive, so the literature is primarily limited to social factors that can be identified and measured within EHRs or administrative databases.

The primary goal of COPD management is to optimize therapies to reduce symptoms and the risk of exacerbations [5]. While nebulizer therapy targeted to certain high-risk COPD patients may improve the transition from hospital to home, specific patient and social factors contribute to difficulties with COPD management. Currently, there is limited literature describing the experiences of COPD patients using and adhering to nebulizer therapy, particularly during care transitions. Understanding the COPD patient’s experience with nebulizer treatment at the transition of care could lead to more effective management of their condition and ultimately improve clinical outcomes. The objective of this study was to describe underlying COPD patient and social factors contributing to practical difficulties with nebulizer use at the transition of care from hospital to home.

## Methods

### Study design, participants, & recruitment

This was a qualitative study using semi-structured, one-on-one interviews with COPD patients recently discharged from a hospital on nebulizer therapy. Eligibility criteria were: 1) patients at least 40 years of age, 2) a current COPD diagnosis, 3) recently discharged to home from an inpatient hospital stay, and 4) currently on scheduled or as-needed nebulizer therapy. Non-English-speaking patients or those with a diagnosis of cognitive impairment were excluded. Subjects were recruited between September 2020 and July 2022 from a family medicine practice group and hospital affiliated with the University at Buffalo. Patients were identified either during their hospital stay or using a discharge notification roster provided monthly by the practices. One of the study investigators (either AAF or DMJ) would follow up with subjects, confirm eligibility, and obtain verbal consent. The 30 – 45-min interviews were conducted telephonically, audio recorded and professionally transcribed. Transcripts were not returned to participants for comment or correction. Completion of the interview marked study completion for the participant, who received a $50 Visa gift card. The University at Buffalo Institutional Review Board (IRB Protocol #3792) approved the study protocol.

### Data collection

At the beginning of the interview, subjects completed a sociodemographic and medical history survey including demographics, prior health care utilization, and socioeconomic status. The COPD Assessment Test (CAT) was used to assess and quantify the impact of COPD symptoms on health status [17]. Semi-structured interviews were conducted by a single investigator (AAF) following an interview guide (online Supplement). The interview guide was developed to include open-ended questions based on the conceptual model by Calvillo-King et al. covering a broad range of social factors and their relationships with readmission [18]. The COPD Foundation’s Root Cause Analysis Tool for readmission [19], a 22-question tool used to help COPD health care providers perform root cause analysis to gauge the contribution of specific factors to an individual patient’s hospital readmission, was also used in interview guide development. Prespecified topics were further informed by a literature review and included patient discharge experience, nebulizer education received at discharge, patient self-description of nebulizer use, factors impacting nebulizer adherence, social support, and medication access. The interview guide was iteratively revised throughout the interviewing process, allowing exploration of emerging topics from different perspectives. The interviewer was given flexibility to have patients elaborate on a topic based on their response through follow-up questions. Data collection continued until the dataset was deemed to be approaching thematic saturation.

### Data analysis

A confidential service transcribed each qualitative interview verbatim, and transcripts were reviewed for accuracy and completeness by the study investigators. Following a constant comparative method, transcripts were repeatedly read and reviewed to achieve familiarity [20]. A thematic analysis of transcripts was performed using an inductive and deductive approach [21]. Preliminary codes identifying emergent themes were open coded by two team members (SR, AAF) through simultaneous reading of each transcript. The study team met frequently to review and discuss transcripts (SR, AAF, DMJ). New codes were added as data not fitting a pre-existing code were encountered. Once the coding framework was finalized and agreed, AAF formally coded all transcripts followed by independent coding review of randomly selected transcripts by DMJ. Codes were subsequently organized into themes and sub-themes using both deductive and inductive practices. Thematic saturation was obtained after interviewing 21 patients. QSR’s NVivo 12 qualitative analysis software (QSR International, Doncaster, Australia) was used for electronic coding, managing data, and generating reports of coded text for analysis. The authors followed the consolidated criteria for reporting qualitative studies (COREQ) guidelines for reporting qualitative research [22]. 

## Results

A total of 46 patients were identified as eligible for the study and 21 patients were recruited. We were unable to reach the 25 remaining subjects after three attempts.” The average age was 64 ± 8.4 years, 13 (62%) were female, and 16 (76%) were White (Table 1). Almost all subjects (95%) used government-funded insurance (Medicare, Medicaid, or both), ten (48%) subjects were unemployed at the time of interview, and most (95%) had an annual income less than $37,999. Subjects reported having multiple comorbidities, the most common being depression (71%), hypertension (62%), and hyperlipidemia (48%) (Table 2). Nine (43%) patients reported currently smoking, 11 (52%) reported previously smoking, and 1 (5%) patient reported never smoking. Of the 9 patients currently smoking, 8 (89) reported smoking less than 1 pack-per-day (PPD) and 1 (11%) reported smoking 1 to 2 PPD. Among the 20 total smokers, 18 (90%) reported smoking for at least 30 years and 2 (10%) reported smoking for 20 to 30 years. On average, subjects took at least 14 regular medications, and a majority of subjects (62%) had at least four COPD exacerbations in the previous year. The subjects had an average CAT score of 26 ± 8, deemed “high” impact, i.e., COPD stopped the patients from doing most things that they wanted to do with significant room for management improvements [17].

**Table 1:** Participant demographic and socioeconomic characteristics

	***N* = 21**
**Age (mean ± SD)**	64 ± 8.4
**Sex**	
Female	13 (62)
Male	8 (38)
**Race**	
White	16 (76)
Black	5 (24)
**Highest level of education**	
Undergraduate degree	5 (24)
Some college	6 (29)
High school/GED	5 (24)
Some high school	5 (24)
**Primary insurance type**	
Medicare	8 (38)
Medicaid	7 (33)
Medicare-Medicaid	5 (24)
Private/commercial	1 (5)
**Marital status**	
Married	6 (29)
Divorced	6 (29)
Single	5 (24)
Widowed	4 (19)
**Employment Status**	
Unemployed	10 (48)
Retired	8 (38)
Employed	3 (14)
**Annual Income**	
< $37,999	20 (95)
$64,000—$99,999	1 (5)

Continuous variables are presented as mean ± SD and categorical variables as No. (%)

*Abbreviations*: *GED* Tests of general educational development, *SD* Standard deviation

**Table 2:** Participant medical history and health care utilization

	***N* = 21**
**Comorbidities**	
Hypertension	13 (62)
Hyperlipidemia	10 (48)
Diabetes	7 (33)
Depression	15 (71)
Chronic kidney disease	3 (14)
Asthma	9 (43)
**Total medications**	14 ± 5
**Years diagnosed with COPD**	10.6 ± 7.6
**COPD exacerbations in past year**	
0	1 (5)
1	1 (5)
2	2 (10)
3	4 (19)
4	4 (19)
> 5	9 (43)
**Hospitalizations in past year**	
1	7 (33)
2	4 (19)
3	5 (24)
4	2 (10)
> 5	3 (14)
**History of respiratory infection hospitalization**	
Yes	16 (76)
No	5 (24)
**Smoking status**	
Current smoker	9 (43)
Former smoker	11 (52)
Never smoked	1 (5)
**COPD assessment test score**^**a**^	26 ± 8.3
**COPD assessment test score impact**^**a**^	
Very high	8 (40)
High	6 (30)
Medium	5 (25)
Low	1 (5)

Continuous variables are presented as mean ± SD and categorical variables as No. (%)

*Abbreviations*: *COPD* Chronic obstructive pulmonary disease, *SD* Standard deviation

^a^Includes 20 of 21 patients. One patient did not respond to a question regarding breathlessness, thus the score and impact could not be calculated

**Fig. 1:**
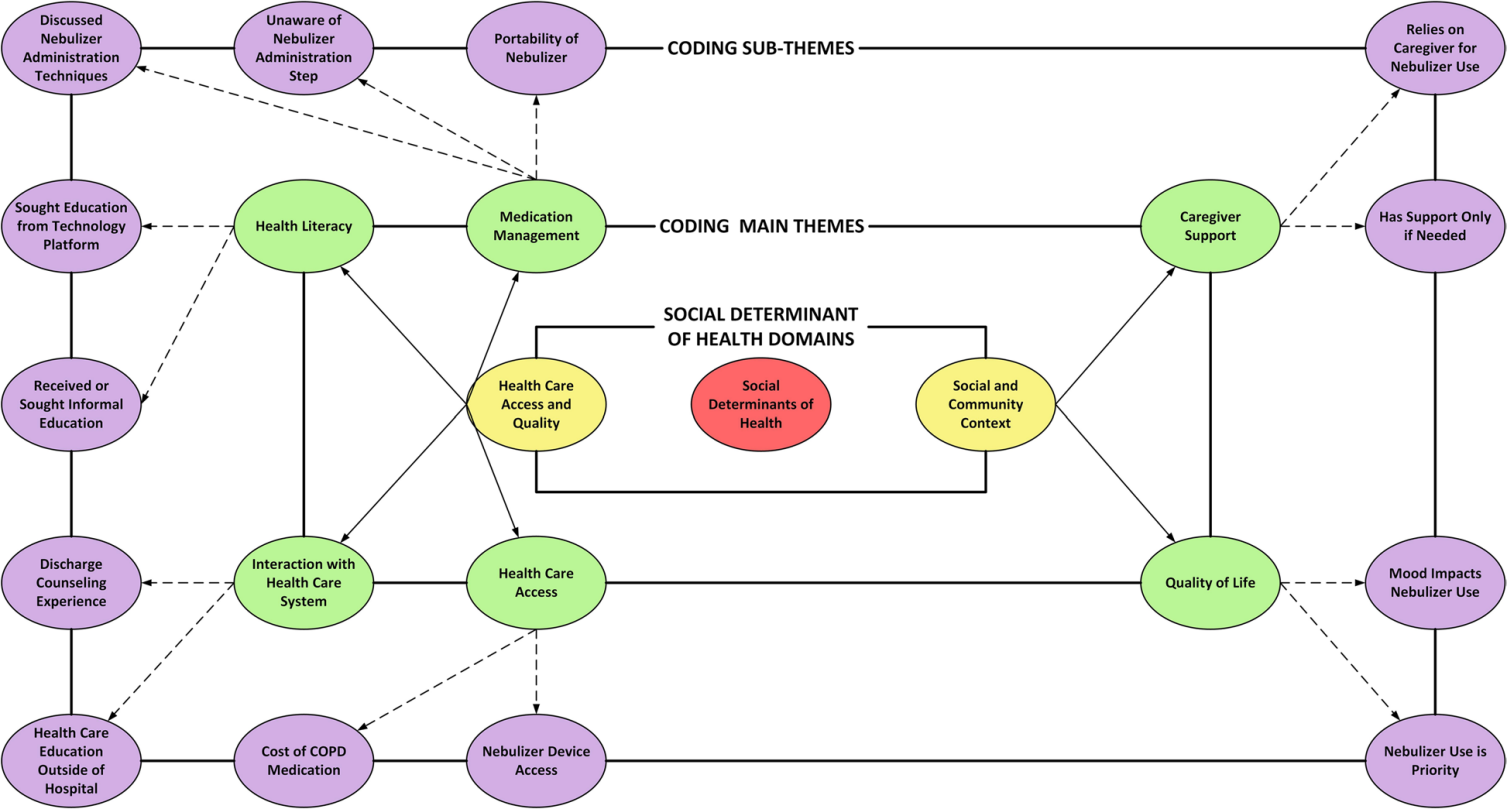
Thematic coding scheme of qualitative interviews. While each code is interrelated, our main focus at the center was social determinants of health (SDoH). In this study, we focused on the primary SDoH domains of “health care access and quality” and “social and community context”. The main themes identified were medication management, health literacy, interaction with the health care system, health care access, quality of life, and caregiver support. The outer ring represents the sub-themes identified for each main theme

**Fig. 2:**
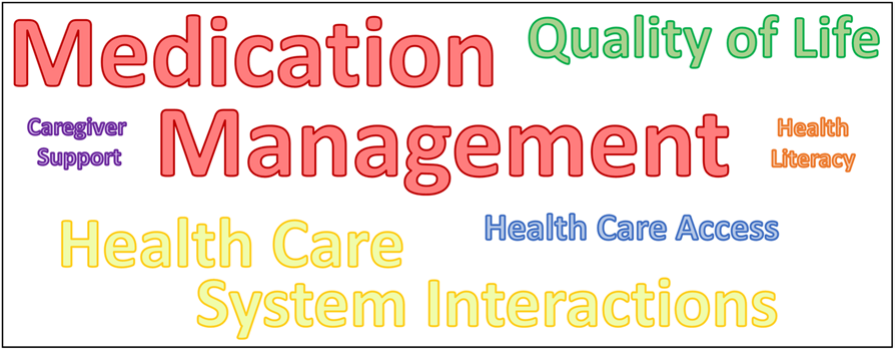
Word cloud analysis of the main theme coding frequencies. Each main theme was coded in the transcripts each time it occurred. The size of the text in the word cloud for each main theme correlates with the number of times each main theme was coded

Six themes were identified from the interviews including medication management, health care system interactions, quality of life, health care access, caregiver support, and health literacy. Within each theme, we identified subthemes based on common discussion points that arose within the interviews. Figure 1 presents an overall coding schematic including the themes and sub-themes, which shows that the major social determinant of health needs identified were “health care access and quality” and “social and community context”. Further identifying the main coding frequencies with a word cloud analysis (Fig. 2), the major coding frequencies from the interviews included medication management, health care system interactions, and quality of life.

### Medication management

Medication management was identified as responses based on the preparation, administration, clean-up, or maintenance of nebulizer therapy. Three sub-themes included nebulizer administration techniques, unaware of nebulizer administration steps, and portability of the nebulizer. Across almost all interviews, patients could appropriately identify, set up, and utilize their nebulizer treatment without difficulties. However, at the same time, there were inconsistencies in how patients were or were not cleaning their nebulizer after each use and replacing nebulizer parts on a regularly scheduled basis. Patient 8 was asked how often they changed their nebulizer parts and stated, “Okay, and how often should I do that? How often should I change out the mouthpiece and the tubing?”.

Many patients did not view portability of their nebulizer as an adherence concern. Most patients who initially had a portability challenge were able to adapt and access portable nebulizer devices. For instance, Patient 4 indicated, “Well, I've only used it [a portable nebulizer] a couple of times. I basically bought it so that I could go camping. You know, for times that I was out. Because it's pretty small. I can carry it around in my coat pocket.” Additional representative interview quotes are provided in Table 3.

**Table 3:** Representative patient interview quotes by theme

**Theme**	**Representative quotes**
**Medication management**	I've got this little bag I put in the dishwasher, and you can put it [nebulizer parts] in the dishwasher, and I just stick stuff in the dishwasher and wash it
	It’s actually very– I thought it was going to be so much more complicated than what it was actually. The one that they gave me is very easy to use
	We didn't know you were supposed to change the mask, and where do we get another mask?
	Well, we [provider and patient] determined a protocol of care when I feel like I'm having one [COPD exacerbation] coming on that ordinarily would send me to the ER. First use the albuterol inhaler, the rescue inhaler twice, and if that doesn't work get home if I'm out or just start up my nebulizer and do one of those treatments, and if it still doesn't work get my butt to the hospital
**Health care system interactions**	My doctor, I don't feel that he has the time. I feel like you're in and out, and I just don't think he’s a teacher
	I know that there was a lot of different things about how ill I was, but nobody discussed anything with me that I can remember
**Quality of life**	I'm sure that there has been a time or three when I wasn't practicing good self-care, but it's the exception and not the rule. Do you know what I mean? If the situation were dire enough there would be no question. I'd take the treatment. But if I think that it's rather minor and I'm– because I still live with PTSD. That's major milestone for me to be able to practice continuous self-care. It's part of the symptoms, it's part of the problem, and working through those things is always gonna be part of the process
	[Regarding mood interfering with COPD treatment] Yes, it has. You know I'm trying to, like my granddaughter was getting over COVID, it's just sometimes a lot of things get stressful, and sometimes I'm just too exhausted as the day is over to do anything. I've done it with that. You know I have a daughter that was murdered, she was murdered September, so her death date is coming up and sometimes you know that get to me where it just I go into a little depression, or, you know it's just certain things
**Health care access**	No, it’s [nebulizer and COPD medication] all fully covered by insurance, so
	Not really, I think it's, with my co-pay it's manageable
	I do want to let you know too when I was in the hospital, they did give me a new nebulizer
**Caregiver support**	No, I do it myself. She’s [patient’s mom] just there in case something happens
	He [patient’s son] knows exactly how to use it. If I can't get to it, like I'll explain to you. On like the week before Thanksgiving I was having an attack and he knew it and I was upstairs, and my own son, it was, this is odd, my hands clamped shut where I could not open my fingers up or my thumb, I could not open my hands up at all. So it's a good thing that he knew how to use that because he got it all set up and he gave me the nebulizer treatment and it didn't do anything and then we did another one and then he drove me to the hospital
	Well, I don't walk, that's one problem I have, so it has to be brought to me. We've got it set up next to my chair, and, unfortunately, I hope to be able to walk someday, but I don't know if that'll ever occur… She [patient’s wife] gets everything ready, makes sure I do it, you know, and she cleans it up, and we get it ready for the next one
**Health literacy**	[Referring to knowing how to use the nebulizer] Well, no, in the end stages of my mother's life my sister and I were her caregivers. We pretty much had beaucoup experience with that kind of thing, and it was all known to me
	[Referring to knowing how to use the nebulizer] Because my grandkids have asthma, and they have to get on machines

### Health care system interactions

Health care system interactions were commonly discussed in the patient interviews. This theme was identified as any interaction between a health care professional and the patient or caregiver. We further identified two subthemes: discharge counseling experience and health care education beyond the hospital setting. There were many inconsistencies in discharge counseling delivery methods and the patient-friendliness of the materials provided. Regarding their discharge paperwork, Patient 3 stated, “I know that they [hospital providers] gave me some paperwork that I didn't really read at all, I didn't, I just haven't had time. I mean, I got the paperwork right beside me, but I don't know what I was supposed to read.”

Upon hospital discharge, most patients were able to connect with their primary care provider; however, the interaction reported with the provider varied. For instance, Patient 2 said, “My doctor, I don't feel that he has the time. I feel like you're in and out, and I just don't think he’s a teacher.” However, Patient 11 had a more positive interaction with their primary care provider. This patient said, “…I talked to him [primary care provider] at least once a week, and then one day he came over to visit me at the house to see how I was doing.”

### Quality of life

Most patients discussed quality of life, identified as patient perceptions of how their COPD or nebulizer therapy impacted their daily life. Two subthemes were identified, including patients prioritizing their nebulizer use and how their mood impacted nebulizer therapy adherence. Almost all patients reported that they prioritized their nebulizer therapy over other daily activities. Patient 10 stated, “No, if I have to use a nebulizer that takes priority.” Another patient echoed a similar sentiment, “I don't care what mood I'm in. I don’t care what's happening, man. That’s [the] first thing I do in the morning, take my medication and do what I have to do for my illness.” However, some patients did report mental health conditions and negative changes in mood impacting their drive to maintain nebulizer therapy adherence.

### Health care access

Health care access was identified as an interaction with the health care system that promoted or impeded nebulizer therapy adherence. Two additional subthemes related to access included COPD medication cost and nebulizer device access. Interestingly, many patients reported that their COPD medications and nebulizer device were affordable. A few patients reported barriers to obtaining a nebulizer device related to insurance coverage and suppliers accepting their insurance. For instance, Patient 12 explained challenges faced in obtaining his nebulizer device, “The three places I went to all told me the same thing, we don't take your insurance, so we can't help you’”.

### Caregiver support

Caregiver support was defined as any interaction between patients and their caregiver(s) where the caregiver may assist with the nebulizer therapy process. Two prominent subthemes were identified: patient reliance on a caregiver for nebulizer use and the patient having support but only if they asked for assistance. Most patients prioritized independent nebulizer use but had a social support system in place if they needed assistance with treatments. One patient relied on their caregiver to administer nebulizer treatments due to severe mobility challenges. In addition, Patient 3 had a unique situation from other participants, explaining, “Well, I have an aide that comes in that she just walked in, and I have one five days a week, or four days a week, and she'll help me, if I need it when she's here she'll help me. But I can handle it myself, I believe. Sometimes it's very close and it's very scary.” Even though this patient had a scheduled support system, she does fears for being able to appropriately address her symptoms when the support system is unavailable.

### Health literacy

Health literacy was identified as a major theme and was defined as the patient’s ability to understand their nebulizer treatment and seek resources when they had a question regarding their therapy. The two subthemes identified were patients seeking education from a technology platform and patients receiving informal education. Most patients reported understanding how to use their nebulizer therapy. Many of these patients reported having other family members or friends who previously assisted with nebulizer treatments and were able to provide patient education. A few patients were unsure of how to perform all the steps in their nebulizer therapy and consulted internet resources to answer their questions. For instance, Patient 2 described her information search, “I think I got most of mine [nebulizer use information] from YouTube, yeah, because they showed pictures and everything.” Separately, Patient 11 indicated, “I guess mostly she [patient’s wife] learned about it from reading up on the internet and talking to the doctor and the druggist.”

## Discussion

In this study, using semi-structured qualitative interviews, subjects described their experience navigating care transitions and managing COPD with nebulizer therapy. Our subjects were primarily those with severe COPD with frequent exacerbations and socioeconomic disadvantages. One significant finding across all subject interviews were the inconsistencies in discharge counseling methods from a hospital visit, with discharge paperwork being described by some patients as overwhelming or challenging to read. Second, patients were able to successfully describe the indication for, set up of, and use of their nebulizer therapy, but there was a large variation in reporting how to clean and maintain their nebulizer equipment. Lastly, all patients reported adaptability in facing daily obstacles in using their nebulizer. All patients highlighted their understanding that nebulizer therapy was important to managing their COPD, and many patients had caregivers available to assist with their nebulizer treatments when needed.

Based on patient responses, health care system discharge counseling practices varied in both method (written, verbal, or both) and depth. When written materials were provided, some patients found them overwhelming or challenging to read, while other patients found them effective. A similar study reported comparable patient discharge experiences from a hospital or skilled nursing facility, with patients facing inconsistencies in care transition processes and social determinants of health issues needing to be addressed after discharge [23]. However, while all the patients in our study reported attending a follow-up appointment with their primary care provider, Jones et al. found that there were racial disparities between patients who did or did not attend follow-up appointments [23]. Furthermore, Horwitz et al. found that while patients perceived discharge care quality and self-rated understanding of discharge instructions as high, actual patient understanding of key aspects of post-discharge care was poor [24]. Consistent with the results of this study, they also found that while written discharge instructions were comprehensive, they were oftentimes not consistently clear [24]. Taken together, these results indicate that there is significant room for improvement in discharge planning, counseling, and written instructions in the current health care system.

When asked to describe a nebulizer treatment from start to finish, study participants were able to successfully describe their symptoms indicating the need for treatment, how to set up and utilize their nebulizer, and the proper inhalation technique. However, patients were inconsistent and often incorrect in describing how they clean and maintain their nebulizer equipment. Some patients said that they used online resources or relied on previous experience from family members or friends rather than reaching out to health care professionals. When Alhaddad and colleagues investigated how patients use their nebulizers at home, they found that patients experienced difficulties with all aspects of nebulizer use and had actually created their own, incorrect strategies to overcome the challenge they faced [10]. Our study demonstrates that the health care system education needs to improve so that patients better understand different aspects of their nebulizer device and to ensure that treatment effectiveness is not compromised.

Most patients did not identify daily obstacles, medical conditions, or mood disorders that impacted their nebulizer use. These patients explained how they prioritized their nebulizer treatments over other activities and adapted to obstacles in real-time. Patients understood the importance of their nebulizer treatments in managing their COPD. In their survey of patients and caregivers about nebulizer use, Sharafkhaneh et al. similarly found that a majority of patients (79%) and caregivers (86%) agreed that the benefits of nebulizer therapy outweighed any difficulties or inconveniences associated with therapy [25]. In addition, 75% of patients and 82% of caregivers also felt that the overall quality of their life (or that of their friend or family member) improved significantly after starting nebulizer therapy [25]. These results demonstrate that nebulizer therapy is a priority for patients with severe COPD and that patients are willing to adapt their daily lives to ensure that they complete nebulizer treatments.

Our study has several important limitations. First, the study faced recruiting challenges due to the COVID-19 pandemic. Indeed, nebulizer use significantly decreased after the start of the pandemic [26], decreasing the population of patients eligible for this study. In addition, four in ten American adults avoided medical care due to concerns about COVID-19 [27]. Second, we did not follow patients longitudinally, which may provide additional insights regarding patient’s social needs and health care utilization patterns. Future studies should consider serial interviews to mitigate this concern. Next, we primarily interviewed severe COPD patients with frequent exacerbations and an array of potential social disadvantages. Patients’ social needs are local to their communities and dynamic in nature; therefore, our findings may be difficult to generalize or transfer to other populations. Social needs screening and navigation programs are becoming more prevalent within different healthcare settings including clinics, hospitals, and pharmacies [28–30]. Successful implementation of these programs will be a priority to overcome social challenges and improve patient care. Finally, the investigator who primarily conducted the interviews was also the lead on qualitative data analysis. Therefore, there could have been unconscious investigator bias, but the primary investigator made every attempt to not deviate from the interview guide. However, we believe these findings provide important insights on the complex interaction between care transitions, healthcare systems, and social care needs among COPD patients using nebulizer therapy.

## Conclusions

The study’s results suggest that the health care system needs to improve and provide consistent, effective discharge counseling for patients leaving the hospital to return home. Furthermore, while patients with COPD discharged from the hospital on nebulizer therapy can access and understand their treatment, they require additional education for nebulizer clean up and maintenance. The interviews also demonstrated patient adaptability and that patients prioritize their nebulizer treatment over almost all other daily activities. Next steps will be to learn more from COPD patients on how the health care system can improve discharge counseling and nebulizer device counseling, so that it improves patient care.

### Supplementary Information

**Additional file 1.**

## Data Availability

The dataset used analyzed during the current study are available from the corresponding author on reasonable request.
