# Supplementary material for: Patient and social factors related to nebulizer use in COPD patients at the transition of care: a qualitative study
Source: BMC Pulm Med. 2023 Sep 23;23:358. doi: 10.1186/s12890-023-02651-w (PMC10517547; doi:10.1186/s12890-023-02651-w)
Supplement: Supplementary file 1 — Additional file 1. [file 12890_2023_2651_MOESM1_ESM.docx]

**Patient and Social Factors Related to Nebulizer Use in COPD Patients at the Transition of Care: A Qualitative Study**

Amanda A. Foster, PharmD, CHW^1,2^; Jennifer Stoll, PhD^3^; Christopher J. Daly, PharmD, MBA^1,2^; Collin Clark, PharmD^1^; Sanjay Sethi, MD^4^; David M. Jacobs, PharmD, PhD^1^

**Supplemental Materials**

**Interview Guide**

1. Think back to when you were about to leave the hospital. What sort of written or verbal instructions did you receive at discharge about your COPD medication or nebulizer?
2. *[for patients who have been readmitted in 30 days]*: Describe any major health or life events that happened between the time you were discharged and when you were readmitted.
3. [*all patients]:* How did you follow-up with your [primary care provider once you were discharged from the hospital? (face-to-face, telehealth, phone call, etc.)
4. Think back to when someone informed you about using the nebulizer…what were the circumstances? Did they provide enough information? Who helped you or informed you about using the nebulizer (e.g., nurse, doctor, pharmacist)? How much time did they spend with you to discuss using your nebulizer?
5. Walk me through a nebulizer treatment: beginning with how you know it’s time for one, to how you assemble the nebulizer, to what you do with the equipment when you’re done with it.
   1. Tell me how you learned about the proper inhalation technique. Were the instructions written down or did someone show you (several times)? How do you know if you’re doing it right?
   2. Tell me how you clean your equipment.
      1. Tell me about your experience in taking apart and putting together back the nebulizer? What did you find easy about it? What did you find challenging about it?
   3. How often do you replace your facemask/mouthpiece, tubing, filters?
6. Have you ever had questions or were unsure about how to use your nebulizer? What did you do? Who or what helped you?
7. Do you ever have to stop your nebulizer treatment early due to interruptions or things happening in your household? Tell me more about that.
8. Is there anything about your daily routine or home life that makes using your nebulizer difficult? Tell me more about that.
9. Has portability of your nebulizer ever been an issue?
10. Do any of your other health conditions (e.g., arthritis, vision problems, nerve or muscle problems) interfere with your ability to use the nebulizer? *If answer is yes*: Could you give me an example of what you mean by that?
11. Does your mood or how you feel about yourself interfere with your motivation to treat your COPD as directed by your provider?
12. Is there anyone in your household or anyone who visits you who helps you with your nebulizer? What sorts of things do they help with?
13. Has cost of equipment or the COPD medication ever been a problem or caused you to refrain from treating your COPD?
14. Have you ever used a different device for your COPD than the one you’re using now? What prompted the change? How does the nebulizer compare with the previous device?
